# Inverse Design Tool for Ion Optical Devices using the Adjoint Variable Method

**DOI:** 10.1038/s41598-019-47408-w

**Published:** 2019-07-30

**Authors:** Lars Thorben Neustock, Paul C. Hansen, Zachary E. Russell, Lambertus Hesselink

**Affiliations:** 1 Stanford University, Department of Electrical Engineering, 226 Serra Mall, Stanford, 94305 CA USA; 2 Ion Innovations, 3815 Courtside Terrace, Norcross, 30092 Georgia USA

**Keywords:** Electronics, photonics and device physics, Design, synthesis and processing, Applied physics

## Abstract

We present a computer-aided design tool for ion optical devices using the adjoint variable method. Numerical methods have been essential for the development of ion optical devices such as electron microscopes and mass spectrometers. Yet, the detailed computational analysis and optimization of ion optical devices is still onerous, since the governing equations of charged particle optics cannot be solved in closed form. Here, we show how to employ the adjoint variable method on the finite-element method and Störmer-Verlet method for electrostatic charged particle devices. This method allows for a full sensitivity analysis of ion optical devices, providing a quantitative measure of the effects of design parameters to device performance, at near constant computational cost with respect to the number of parameters. To demonstrate this, we perform such a sensitivity analysis for different freeform N-element Einzel lens systems including designs with over 13,000 parameters. We further show the optimization of the spot size of such lenses using a gradient-based method in combination with the adjoint variable method. The computational efficiency of the method facilitates the optimization of shapes and applied voltages of all surfaces of the device.

## Introduction

Electromagnetic fields have been applied to accelerate, guide and focus ions and electrons for over 100 years, with applications as far-ranging as vacuum tubes, electron microscopes, terahertz emitters, mass spectrometers and high-energy particle accelerators^1,2^. The (nonrelativistic) motion of charged particles obeys a nonlinear coupled system of equations, composed of the Laplace equation for the electrostatic potential and a combination of Newton’s and Coulomb’s laws for the equation of motion. Neither equation can be solved in closed form for any but the simplest devices. Instead, engineers and physicists rely on numerical modeling to understand and design ion optical devices^3^. To optimize a device, most commonly used approaches must analyze an extensive number of devices, which can be prohibitively slow. To avoid the effort of such an extensive analysis, the method introduced here computes the effects a design parameter variation, such as device dimensions or applied voltages, has on the physical behavior of ion optical devices. These effects are called design sensitivities and can be calculated efficiently using the adjoint variable method (AVM). Quantitative knowledge of the design sensitivities not only enables local optimization of devices, but also aids physical understanding. It helps visualizing how design parameters, such as each point on a surface, should be perturbed to enhance device performance. We present the application of AVM for the optimization of electrostatic ion optical, or more generally charged particle optical, systems. This is, to the best of our knowledge, the first application of AVM to ion optical device optimization and the first derivation of a discrete adjoint system for charged particle devices. In this work, we describe how the computational efficiency of the method allows us to not only analyze and optimize the key dimensions but also the shape and applied voltages of all surfaces of a design.

The described method easily integrates into the design process for ion optical systems, because it uses the discrete adjoint approach and is based on established simulation models^4^. With AVM, the design process not only contains numerical modelling of a device, but also a sensitivity analysis. In a sensitivity analysis, all design sensitivities of a device are computed. The geometry of a device is represented numerically first. Then, a simulator based on the governing differential equation predicts the electromagnetic fields with a rigorous technique such as the finite element method (FEM)^5^. Next, the equation of motion for the charged particles is solved, most commonly with a Runge-Kutta method or a Störmer-Verlet method (SVM)^6^. We can then calculate the design sensitivities by solving another set of systems posed using AVM.

Adjoint design sensitivity analysis has enabled design optimization in structural engineering^7–10^, aeronautics^11–13^, microwave engineering^14–17^, magnetic materials^18^ and photonics^19–30^. Here, we detail how to use AVM for any electrostatic ion optical design problem. This approach is extensible to RF and magnetic systems as well^31^, and to systems with space charge and other particle-particle interactions. We implemented a demonstration tool for electrostatic ion optical devices in 2D and cylindrical coordinates. We further demonstrate the AVM method with the optimization of N-element freeform Einzel lens systems, an example of ion optical devices. We provide a computational tool to design and optimize arbitrarily-shaped charged particle optics parts. The computer-aided optimization of shapes for ion optical devices complement the progress in additive manufacturing of metal parts, overcoming fabrication constraints that can prohibit the usage of inverse design tools in areas such as photonics.

Einzel lenses are used in different form and settings for nearly all ion and electron beam lensing applications, ranging from imaging^32^ to time-of-flight mass spectrometry^33^. They focus ions in flight by manipulating the electrostatic field along the ion trajectory. Einzel lenses are easy to manufacture and align, and their spherical aberrations are independent of the charge-to-mass ratio of the particles^34^. Einzel lenses are usually designed on a case by case basis for the application at hand. Yet, even with a few variable parameters, *e.g*. only the lens elements’ spacing and applied voltages, there can be hundreds of possible designs to consider as the optimal design for any application. Thus, despite Einzel lenses being one of the simplest ion optical devices, they remain difficult to optimize. In the past, a common strategy to mitigate the cost of analysis of many different designs was to use faster, but more specialized and approximative, numerical models, a compromise that pitted speed against ability to accurately model the physics of arbitrary devices^34,35^. Today it has become more common to employ brute force, modeling many devices in parallel on computer clusters and using slowly-converging optimization methods such as genetic algorithms^36,37^. With AVM, we can optimize Einzel lenses with a fast-converging algorithm, which requires low execution time. This can revivify the computer-aided optimization of ion optical lenses and related devices with even more complex geometries.

## Results

The calculation of accurate gradients lays at the core of any optimization routine and computer-aided design. The strength of the method detailed here is its ability to compute gradients accurate to machine precision. For ion optical systems, this is of high importance. Even small differences in field strength can cause an ion or electron to take a different trajectory. To ensure accurate gradients, we implemented full in-house simulations for the forward and adjoint FEM and SVM applying the discrete adjoint method. To test for accuracy, we compared the results of a demo forward system with an analogous SimIon simulation and obtained very good agreement (less than 1% error)^38^. We further tested the gradient calculation by solving perturbed systems and calculating finite differences. All appreciably nonzero gradient components agree with finite differences to within 1%. This agreement is extremely high and cannot be achieved with continuous adjoint approaches, yet a requirement to effectively optimize an ion optical system. Subtle changes in the geometry of such systems can have a significant effect on the electrostatic field distribution and ion trajectories, thus slightly inaccurate gradients would cause a diverging objective function during the optimization procedure.

**Figure 1:**
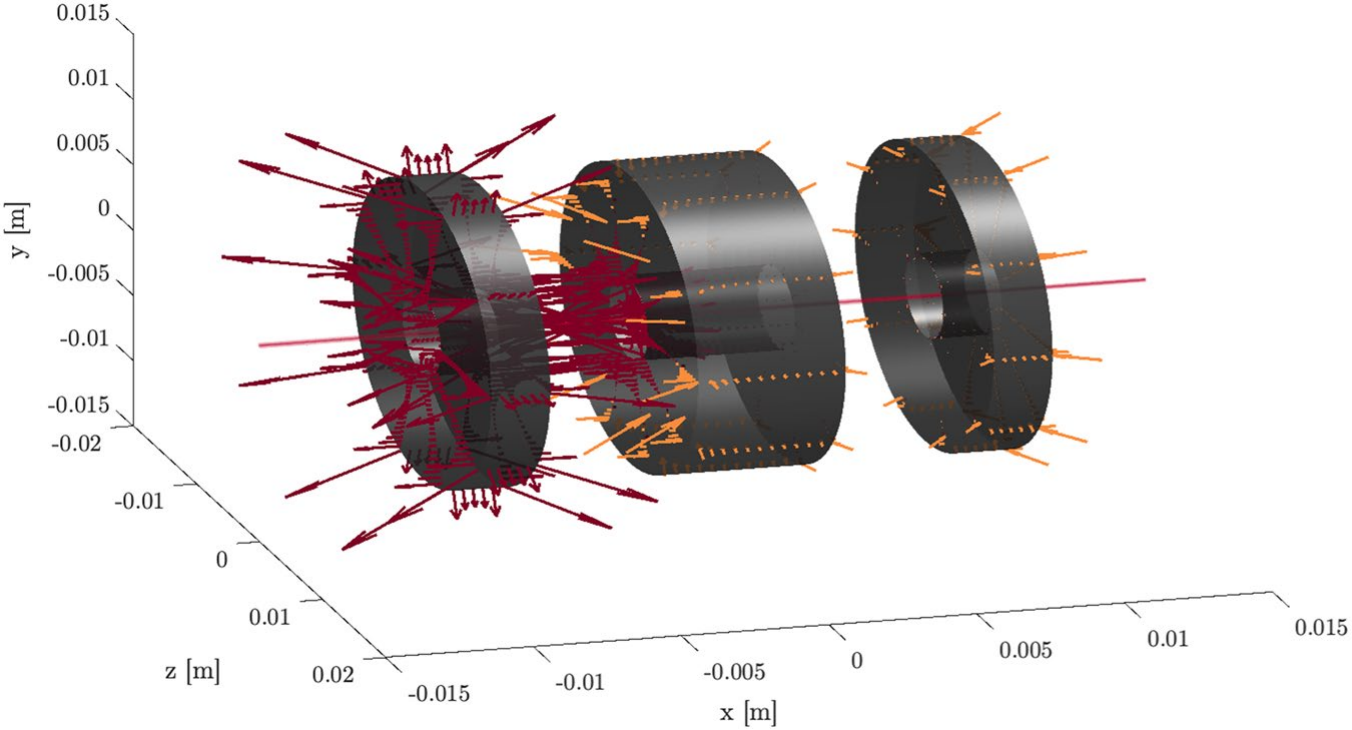
Sensitivity analysis of an Einzel lens: Orange arrows push while red arrows pull the shape in the direction of a more optimal shape.

In Fig. 1 we show the result of a sensitivity analysis of an electrostatic Einzel lens’ surface graphically. We conducted a sensitivity analysis of free-form *N*-element Einzel lens systems with over 13,000 moveable points. Using such a high number of degrees of freedom demonstrates the ability of the method to optimize highly-parameterized systems and shapes. We furthermore optimized several Einzel lenses with up to seven freeform elements.

### Setup

**Figure 2:**
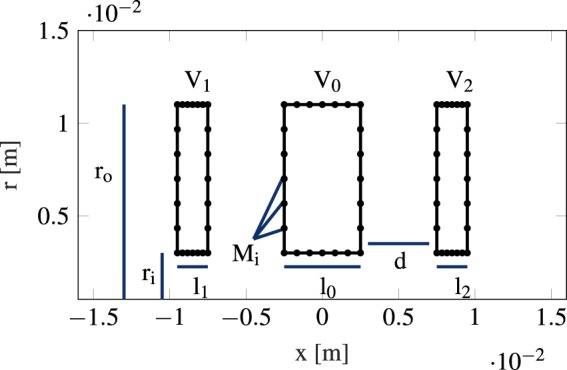
Upper half of the cross section of the initial design of a three element lens. The x-axis is the symmetry axis.

The Einzel lens shape as it is set up for an optimization is shown as a cross section in Fig. 2. This figure shows the upper half of a cylindrically-symmetric Einzel lens with the x-axis as symmetry axis. The total simulation space with a rectangular cross section and Neumann boundaries is 180 mm in length and 135 mm in height. The simulated Einzel lenses have three or more tubes with imposed voltages. The initial geometries are described in Table 1.

**Table 1:** Initial Einzel lens shape parameters (clc. Fig. 2).

Parameter	Description	Value
*V*_*i*, *i*>0_	voltage of rear elements	0 kV
*V*_0_	voltage of center element	29 kV
*l*_0_	length of center element	5 mm
*l*_*i*, *i*>0_	length of rear elements	2 mm
*r*_*o*_	outer radius	11 mm
*r*_*i*_	inner radius	3 mm
*d*	element spacing	5 mm

**Figure 3:**
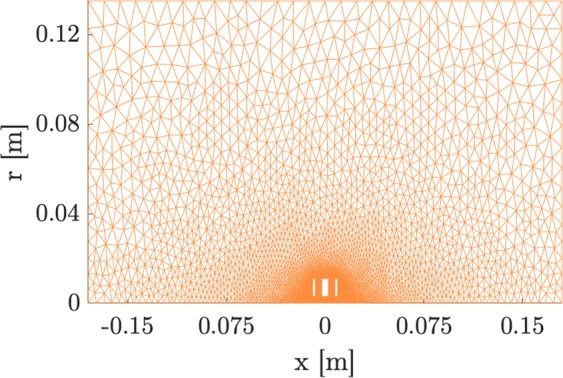
Simulation space, the upper half of a cross section of the geometry, showing the mesh.

In the electrostatic calculation, we chose Lagrange polynomials of order 5 to ensure accurate gradients. We interpolate the potential field onto a Cartesian grid with spacings of Δ*x* = 0.29 mm and Δ*y* = 13.5 nm. The incoming particle beam of radius 0.2 mm is comprised of 100 equally spaced particles flying with an initial kinetic energy of 30 keV in the *x*-direction and with a mass of 5.1477 ⋅ 10^−26^ *kg*, representing Ga atoms. We chose the most general case of equally-spaced particles as any other beam shape can be achieved by weighting the particles in this beam profile. The SVM calculates the particle trajectories over a time span of 278 ns using 1500 time steps. The desired focal point lies on the *x*-axis 50 mm away from the center of the middle electrode. We tested the simulated system for convergence of the gradients by increasing the input parameters until changes to the gradient become marginal. The resulting mesh is shown in Fig. 3.

### Optimal designs

We simultaneously optimized shapes, positions and voltages of several *N*-element Einzel lens systems with freeform shapes. The shapes of the electrodes are polygons of *N* points; the points are parameterized as *x*_*i*_ = *x*_*c*_ + *r*_*i*_cosΔ*θ*_*i*_, *y*_*i*_ = *y*_*c*_ + *r*_*i*_sinΔ*θ*_*i*_. During optimization the electrode centers *x*_*c*_, *y*_*c*_, the boundary displacements *r*_*i*_ and Δ*θ*_*i*_ and the applied voltages are designable, resulting in $3N+2\sum _{i=1}^{N}\,{M}_{i}$ parameters, with *M*_1_, *M*_2_,..., *M*_*N*_, describing moveable points on the surface (see Fig. 2). The step size for the gradient descent is recalculated at each iteration using the Barzilai-Borwein method^39^.

**Figure 4:**
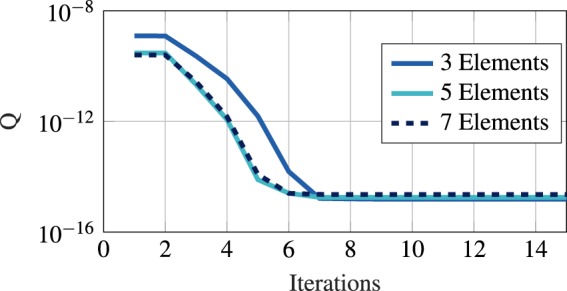
Progression of the objective function during the optimization for selected three-, five- and seven- element Einzel lens systems.

**Figure 5:**
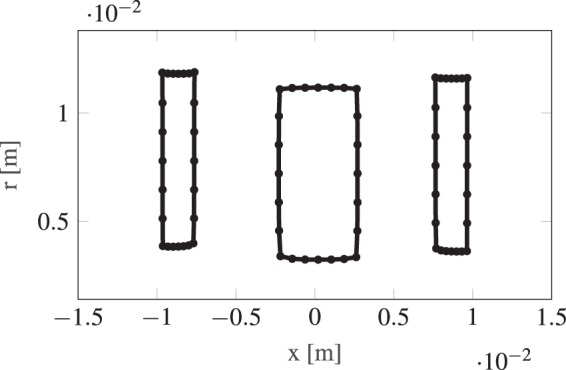
Optimized designs of a three element lens.

**Figure 6:**
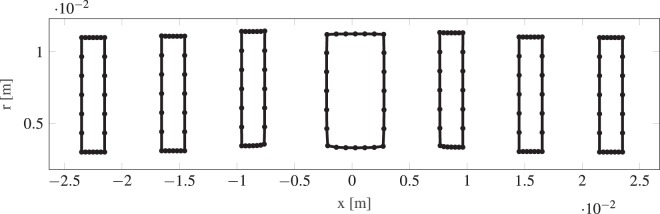
Optimized designs of a seven element lens.

We have optimized several different Einzel lens systems with varying degrees of freedom. In Fig. 4, we show the change of the RMS spot size during the optimization for systems with three, five and seven elements as illustrative examples. Each element side has seven moveable points, a coarse parameterization to limit feature size and allow for easier fabrication. As visible from Fig. 4, only about ten iterations of the gradient-descent method are required for a converged result. At each iteration, a sensitivity analysis using AVM is performed. The optimizations converged with a similar number of iterations independently of the number of elements of the initial geometry. In Figs 5 and 6, we show the result of a three-element and seven-element lens optimizations. The optimized designs include a relative shift of the Einzel lens tubes as well as a curving of the device shape. We reduce the RMS of quadratic spot sizes of our systems from a value of 10^−9^ to 10^−15^, corresponding to a focal spot of about 50 nm.

## Discussion

The optimizations of tested Einzel lens systems remain mostly invariant of the degrees of freedom and number of lens elements. The accurate calculation of the gradient allowed them to converge to the same small spot size as the examples shown in Fig. 4. We thus conclude that we cannot further reduce the spot size. As described by Scherzer’s Theorem^40^, cylindrically-symmetric systems cannot reduce aberrations to zero. Further improvements of the system can be done by breaking the symmetry of the system or through the addition of time varying fields^40^. AVM is applicable to such systems as well.

**Figure 7:**
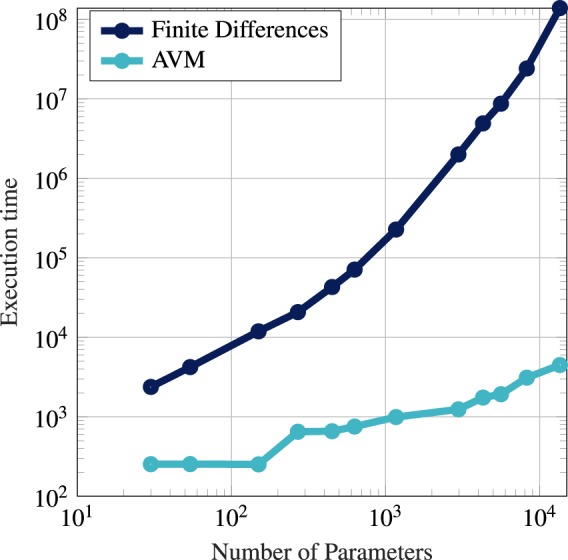
Comparison of runtimes of the adjoint and a finite differences approach to calculation design sensitivities.

**Figure 8:**
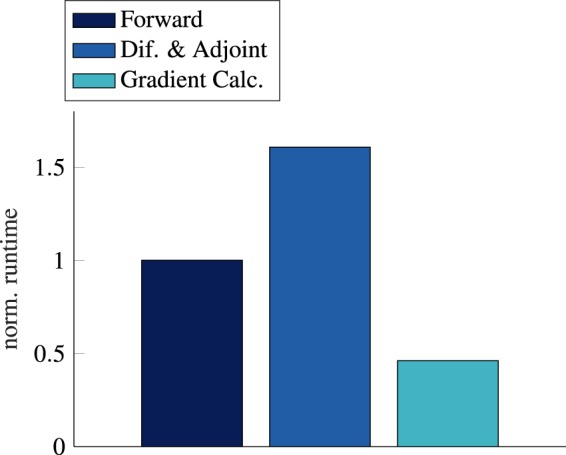
Runtime for a sensitivity analysis separated by its two components. The runtimes are normalized to a forward system solve to account for different machine hardware.

In addition to highly-accurate gradients, high-dimensional sensitivity analysis is the strength of AVM. The execution time of the gradient calculation using AVM is not strongly affected by the dimensionality of the parameter vector. We demonstrate this for Einzel lens systems in Fig. 7. Here, we have conducted a sensitivity analysis for designs with a varying number of design parameters. Even for designs with over 13,000 design parameters, the execution time only moderately increased in comparison to designs with fewer parameters. This increase is mostly due to the growing number of elements in the mesh as lens elements are added. In Fig. 8, the execution time for the sensitivity analysis of a lens with 250 design parameters is broken down into three parts: the forward system, the system differentiation together with the adjoint system, and the gradient calculation. For a single system, the computations to pose and solve the adjoint system generally require a longer execution time than the forward system. The gradient calculation is, as expected, comparably cheap. If the execution time of the forward system increases due to more mesh elements, the execution time for the differentiation and adjoint system will also increase.

In Fig. 7, for further illustration and comparison, we show the execution time of a brute-force finite differences sensitivity analysis obtained through repeating the shared forward calculation. As expected, the execution time scales strongly with the parameter vector dimension in this case. For the case of an Einzel lens device with 13,530 design parameters the sensitivity analysis with AVM runs roughly 30,000 times faster and could become too burdensome to conduct with finite differences unless vast computing power is used. The optimizations using AVM, as shown here, can run on a simple laptop or desktop computer.

Unlike optimization algorithms such as genetic algorithms, gradient descent using AVM is not based on a randomized exploration of the design space. Instead, we rely on a sensitivity analysis. This allows for rapid local optimization. Because the design space for ion optical devices is not convex — many device designs appear optimal compared to their nearest neighbor in design space — we are not guaranteed to find the global optimum^41^. However, the AVM enables the computationally cheap optimization of a diverse variety of initial designs, making it likely that a (near) global optimum will be found.

Sensitivity analysis has applications beyond optimization. With it we can also visualize and quantitatively compare the effects of all design parameters. This allows us to understand which degree of freedom has strong effects on a device design. This physical insight can also be further used for tolerancing. Without the AVM, exact effects of changes in the geometry of the ion optical device to its behavior are opaque to a engineer or physicist as the governing equations cannot be posed or solved in closed form.

The AVM we have shown here allows choosing any arbitrary initial geometry, initial incoming particle distribution as well as objective function. Any of these can be changed in the setup and a optimization program can be run. Thus, many other ion optical devices with more intricate geometries or particle beams than an Einzel lens can be optimized using AVM. Since the parameter vector for the design optimization of such geometries can be large, it has been shown in other engineering fields that optimal designs can exhibit fine-grained features that would not be achievable by a sophisticated guess or through human intuition^42^. With the work shown here, this is possible for ion optical devices. Recently, advances in additive manufacturing for metal parts made it possible to built nearly any shape, including those with fine-grained features^43^. With computational, computer-aided, design for ion optical devices, the full range of the capabilities of this layer-by-layer production of parts can be seized.

In this paper, we build the first ion optical device design tool based on the discrete adjoint variable method building on FEM and SVM. We applied this tool for sensitivity analysis of Einzel lens systems, quantifying how their performance changes with changes to their shape and applied voltages. We analyze the execution time with respect to the length of the parameter vector and show that longer parameter vectors (up to 13,520) only cause a small increase in execution time, allowing for the analysis and optimization of designs that would be too computationally burdensome (>30,000 times slower) with approaches such as finite differences. We further show how to use sensitivity analysis in combination with steepest gradient descent to built a computer-aided design and optimization tool for electrostatic ion optical devices. The potential applications for a design tool using AVM’s range from electron focusing devices to mass spectrometers to electron source design. The AVM tool here could be added as an extra layer to most conventional ion optics tools, which use the same numerical methods as their basis. Thus, the computer-aided design tool can help engineers or physicists to search, explore and understand the space of device designs by reducing the nearly intractable problem of device design to the usage of the presented tool. In combination with additive manufacturing techniques, computer-generated designs will allow for rapid prototyping of charged particle devices and thus facilitate innovation in charged particle optics.

## Methods

### Physical system of ion optical devices

The trajectories of charged particles in vacuum will bend in an electric field imposed by electrodes at fixed applied voltages. Consider the flight of charged particles through a space Ω. Electric potentials are imposed on electrodes with surfaces ∂Ω. For a particle of charge *q* and mass *m*, its trajectory is determined by Newton’s second law and Coulomb’s law,

1 $$m\ddot{{\bf{x}}}(t)=q{\bf{E}}({\bf{x}}(t)).$$

The electric fields are derived from the electric potential *ϕ*, a solution to the Laplace equation,

2 $$\begin{array}{cccc}{\nabla }^{2}\varphi  & =\, & 0 & {\rm{on}}\,{\rm{\Omega }}\,\\ \varphi  & =\, & {V}_{{\rm{applied}}} & {\rm{on}}\,\delta {\rm{\Omega }}.\,\end{array}$$

In this paper, we assume that only the electrostatic field from the electrodes exerts a force on the particles and neglect the electromagnetic field caused by the particles themselves (e.g. from space charge). This approximation is accurate for devices operating in the low beam current limit.

### Optimization using a sensitivity analysis

The performance of an ion optical device can be altered by varying its design parameters. All the changeable parameters, such as sizes and applied voltages of a device design are concatenated into a vector **p**. A performance benchmark to be optimized is called an *objective function Q*(**x**), *e.g*. the focus quality, depending on the device behavior **x**. An optimization attempts to find the design of the device which minimizes *Q*(*x*), but still obeys *constraint functions f*_1,2,3,..., *n*_(**x**), such as physical equations and boundary conditions. The setup to find such an optimal design can be summarized in an *optimization program*,

3 $$\begin{array}{ll}{\rm{minimize}} & Q({\bf{x}}({\bf{p}}))\\ {\rm{subjectto}} & {f}_{1,2,3,\mathrm{..},n}({\bf{x}})=0.\end{array}$$

Global optimization methods, such as simulated annealing^44^, guarantee an optimal solution to such a program but converge very slowly. Faster convergence is achieved by applying local optimization techniques. They only reach local optima, unless the optimization problem is convex^41^. Local optimization techniques such as *steepest gradient descent*, or *gradient descent*, require knowledge of the *full gradient* of *Q*(**x**(**p**)) at each step of the algorithm. For ion optical devices, the gradient describes how the device performance changes under perturbations to its shape, dimensions and operating conditions.

#### Optimization of einzel lenses

**Figure 9:**
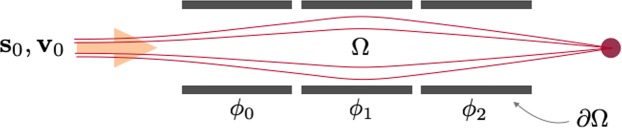
An ion beam focused with initial positions **s**_0_ and velocities **v**_**o**_ by a three-element Einzel lens.

A three element Einzel lens is show in Fig. 9. Collimated charged particles enter the lens from the left, are redirected by the electric fields between elements and are focused to a small spot. The size of this spot determines the quality of the lens. A smaller spot has a smaller objective function *Q*(**s**). The spot size can be converted into the spherical aberration coefficient, commonly used to quantify the quality of a focus, if spherical aberrations are the dominant aberration. Here **s** describes the particle trajectories.

For a single particle, *Q* can be defined as:

4 $$Q=\parallel \hat{{\bf{s}}}-{{\bf{s}}}^{\ast }{\parallel }_{2}^{2}\,\mathrm{with}\,\hat{{\bf{s}}}={\bf{s}}\,{\rm{at}}\,{s}_{1}={s}_{{\rm{f}}}$$

where s* describes the desired focal point at a focal plane defined by the position *s*_1_ = *s*_f_ . For a beam of several particles, we calculate the RMS of *s* − s^*^ over all particles.

**Figure 10:**
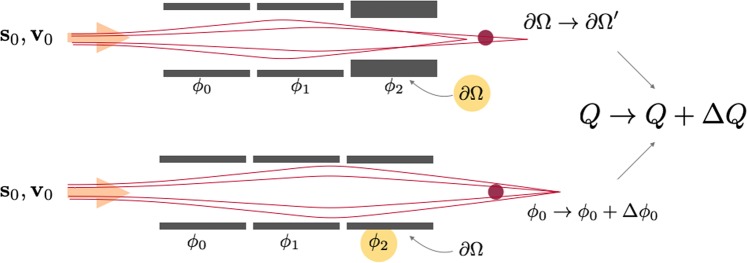
Design sensitivities for an Einzel lens system. Top: sensitivity of Q to shape changes. Bottom: sensitivity of Q to voltage changes.

The partial derivatives of *Q* under changes in the element geometry and voltage are the design sensitivities of the Einzel lens illustrated in Fig. 10.

### Adjoint variable method for ion optical devices

**Figure 11:**
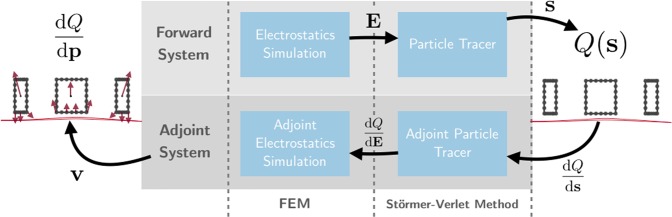
Flow diagram of the sensitivity analysis and optimization of ion optical devices using AVM. The resulting design sensitivities are visualized as arrows.

The adjoint variable method poses equations for the efficient calculation of design sensitivities. Because the governing equations of ion optical devices cannot be solved in closed form, there is no closed form for their gradient either. The gradient can be approximated numerically by finite differences, which requires solving a *forward system* composed of the physical equations with the current parameter vector and, in addition, at least one extra system simulation per design parameter. If the device design is described by a system subject to N parameters, N + 1 full system simulations are required to calculate the gradient by this brute force technique. Yet, a system solve is a relatively costly operation and the number of design parameters can become virtually infinite, when not only the dimensions of a device or applied voltages, but also points on the device’s surfaces are parameterized. In this case, a sensitivity analysis can be conducted by using AVM. The calculation of the gradient with this method only requires solving one additional system after the forward system, the *adjoint system*. The workflow for this technique is described in Fig. 11. This suffices for any number of design sensitivities, so calculating hundreds or thousands of sensitivities scarcely takes more time than calculating one^45^.

To conduct a sensitivity analysis of an ion optical system and optimize it, we first calculate the forward system by simulating the electrostatic field and, subsequently, the particle trajectories. By evaluating the objective function *Q*(*s*) and its derivative $\frac{{\rm{\partial }}Q}{{\rm{\partial }}{\bf{s}}}$, we gain an understanding of how the trajectories would need to be bent to optimize the design [Eq. (15)]. This *direction of error*, the discrepancy between the particle position and the desired focal point $(\frac{\partial Q}{\partial {\bf{s}}}\propto (\hat{{\bf{s}}}-{{\bf{s}}}^{\ast }))$, gets backpropagated into an adjoint particle solver [Eqs (8) & (14)]. We gain an insight into how to change the electric fields to cause the desired behavior, $\frac{\partial Q}{\partial {\bf{E}}}$. Using an adjoint electrostatics solver, we can backpropagate again and calculate how the error depends on different parts of the system, *e.g*. the shape or applied voltage. Since we can analytically determine the influence of the system parameters to the system, we can calculate the design sensitivities $\frac{{\rm{d}}Q}{{\rm{d}}{\bf{p}}}$ using computationally cheap vector-matrix-vector products [Eqs (9) & (16)]. For large system matrices, solving a large partial differential equation (PDE) system is likely to be so much slower than evaluating matrix-vector or vector-vector products that these products can be seen as instantaneous. Thus, the execution time for a sensitivity analysis with AVM is practically *O*(1) as compared to *O*(*N*) with other approaches. An optimization technique using AVM for sensitivity analysis is oftentimes referred to as *inverse design*, since we start the design process with a desired change in outcome and then proceed by calculating the required pertubations to the system.

#### Mathematical formulation of the adjoint variable method

The adjoint variable method makes use of the mathematical concept of *duality*, which describes a relationship between the solutions of two linear systems. We employ this concept to pose a *dual* or *adjoint* system that allows for a efficient gradient calculation^45,46^.

Again let *Q*(**x**) be the objective function operating on the vector **x**. Here, **x** is the numerical solution of a physical model of the system, so its values are state variables such as numerical values of positions, velocities and electric potentials. The simulated physical system, called the *forward system*, can be written as

5 $${\mathfrak{L}}({\bf{x}};{\bf{p}})=0$$

where the function ${\mathfrak{L}}$ expresses all governing equations, boundary conditions and their dependencies on the design parameters **p**. We seek the parameters that minimize *Q*(**x**(**p**)) by gradient descent, so we need the design sensitivity $\frac{{\rm{d}}Q}{{\rm{d}}{p}_{i}}$ for each element *p*_*i*_ of **p**. It follows by the chain rule,

6 $$\frac{{\rm{d}}Q}{{\rm{d}}{p}_{i}}=\frac{{\rm{\partial }}Q}{{\rm{\partial }}{\bf{x}}}\frac{{\rm{d}}{\bf{x}}}{{\rm{d}}{p}_{i}}.$$

While the derivative $\frac{\partial Q}{\partial {\bf{x}}}$ can be found easily, the sensitivity of state $\frac{{\rm{d}}{\bf{x}}}{{\rm{d}}{p}_{i}}$ is the solution of a directly-differentiated system^19^ and is only obtained with effort comparable to solving (5) again. We do so by differentiating (5) to discover a linear system

7 $$\frac{{\rm{\partial }}{\mathfrak{L}}}{{\rm{\partial }}{\bf{x}}}\frac{{\rm{d}}{\bf{x}}}{{\rm{d}}{p}_{i}}=-\frac{{\rm{\partial }}{\mathfrak{L}}}{{\rm{\partial }}{p}_{i}},$$

with a matrix $\frac{\partial {\mathfrak{L}}}{\partial {\bf{x}}}$ commonly renamed^11^ *A*. The crux of AVM is consolidating much of the effort of (6) into the solution of an *adjoint system* that solves for a *adjoint variable* **v**,

8 $${A}^{T}{\bf{v}}=\frac{\partial Q}{\partial {\bf{x}}}.$$

Calculation of the sensitivities is expedited by knowledge of **v**, as it need not be recalculated for each *i*. The derivative components now are easily computed with the vector products

9 $$\frac{{\rm{d}}Q}{{\rm{d}}{p}_{i}}={{\bf{v}}}^{T}(\,-\frac{{\rm{\partial }}{\mathfrak{L}}}{{\rm{\partial }}{p}_{i}}-\frac{{\rm{\partial }}A}{{\rm{\partial }}{p}_{i}}{\bf{x}}),$$

as can be verified by glancing at equations (5), (6) and (8).

All required components for the gradient calculation using AVM can be derived analytically by differentiating the physical equations and implemented directly.

### Sensitivity analysis of ion optical systems

In the case of ion optical devices, the objective function depends only on the particle trajectories; its dependence on design parameters is indirect, mediated by the equation of motion and the Laplace equation, which constrain all designs to obey physical laws. We need to simulate forward and adjoint systems for both of these equations. We can then optimize the ion optical system according to the workflow process outlined in Fig. 11. The simulators are detailed in the following subsections. We implemented fully discrete adjoint systems for both components to ensure accurate gradients to machine precision. To reduce memory requirements and allow our system to run on a simple laptop computer, we calculated equation (9) in a step-wise approach for each FEM element and only using relevant sub-matricies.

#### Electrostatics simulations

The finite element method (FEM) approximates the exact solution of a PDE (partial differential equation), *e.g*. the Laplace equation (2), on a mesh^5,47^. We choose an unstructured triangular mesh, created by the software *gmsh*^48^, for simplicity. Inside each triangle, the field is approximated by a polynomial and represented by its value at a fixed number of points called *nodes* distributed on the corners, edges and interiors of the triangles. We chose Lagrange polynomials as our basis. With these restrictions, the problem can be posed as a linear system:

10 $$\begin{array}{l}M\varphi =f\end{array}$$

wherein the system matrix *M* consists of the discretizations of the Laplace equation and *f* describes the boundary values of the problem. The boundaries can satisfy either a Neumann or a Dirichlet condition. The solution of this linear system, *ϕ*, is the vector of the electrostatic potentials at every node. The values of *ϕ* in combination with the Lagrange basis functions are used to interpolate the values of the electrostatic field onto a 2D cartesian grid of field values as required for the particle tracer.

**Figure 12:**
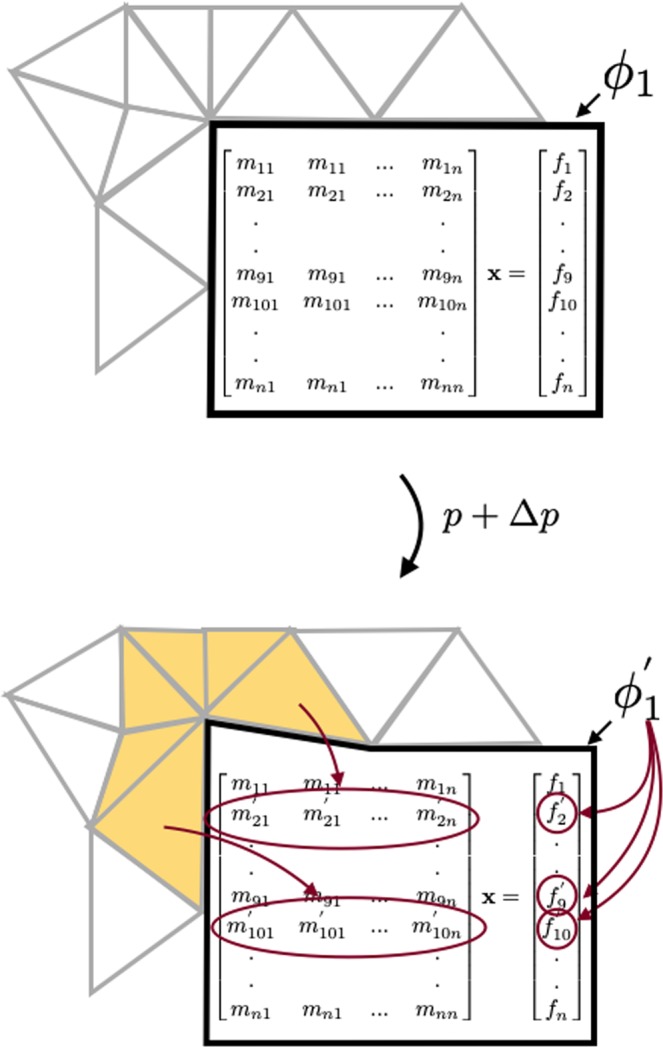
A change of design parameters: Boundary shape changes affect rows of the system matrix. Boundary voltage changes affect rows of the inhomogeneous term.

To differentiate the finite-element method, we study how design changes affect the underlying system of equations. Such changes may cause modifications in shape or changes in the applied voltages. If one corner of an electrode shifts, the edges of all adjacent mesh elements shift. This *mesh deformation* is illustrated in Fig. 12, where the mesh elements affected by the change in shape are marked in light orange. As the mesh deforms, the field nodes — used for the Lagrange basis functions — move with it. Each moving node effects several rows in the FEM system matrix *M*. The imposed potential on electrode surfaces may also be adjusted, causing the inhomogenous term of equation to vary. All these changes to the system have to be accounted for when differentiating the system^5,45,47^. The adjoint electrostatics simulation takes input from the adjoint particle tracer and then calculates the adjoint system variable for the gradient calculation.

#### Particle tracer

The particle equations of motion are solved with SVM^6^. SVM is an iterative method that divides the examined time into *N* time steps of size Δ*t*. Starting with the initial position and velocity of each particle, the method calculates the particle positions and velocities at subsequent time steps,

11 $$\begin{array}{l}{{\bf{s}}}_{n+1}={{\bf{s}}}_{n}+{{\bf{v}}}_{n}{\rm{\Delta }}t+\frac{1}{2}{{\bf{a}}}_{n}{\rm{\Delta }}{t}^{2},\\ {{\bf{v}}}_{n+1}={{\bf{v}}}_{n}+\frac{1}{2}({{\bf{a}}}_{n}+{{\bf{a}}}_{n+1}){\rm{\Delta }}t.\end{array}$$

The acceleration vector **a** is interpolated from the grid of electrostatic field values. After calculating the trajectories, the forward simulation is complete and the device performance can be evaluated.

To find $\frac{{\rm{d}}Q}{{\rm{d}}{\bf{E}}}$, we pose an adjoint SVM system. With the forward solution of the non-linear SVM, we can determine a interpolation matrix $\Im$ used to represent the interpolation of the electric field required to obtain the acceleration vector **a**. The values of the electric field are stored in a vector **E** calculated by the electrostatics solver. The positions of the particles at each time step determine the interpolation weights incorporated in $\Im$ and the indices of the interpolated field values the structure of $\Im$. We write:

12 $${\bf{a}}=\Im {\bf{E}}.$$

We can then write the system of equations using a *movement matrix W* to describe the continuing movement of particles at each time step without added accelerations and an *acceleration matrix W*_*a*_ to introduce accelerations:

13 $$\begin{array}{l}W{\bf{s}}=-\,{{\bf{s}}}_{0}-{W}_{a}{\bf{a}}({\bf{s}})\end{array}$$

Here, the vector **s** contains positions and velocities of the trajectory at all simulated time steps, and **s**_0_ the positions at the initial time step. This system is non-linear, since the vector **a** depends on the particle positions **s** through $\Im$.

Through differentiation of the equations (12) and (13) with respect to the electric field values on a grid, we can pose an adjoint system in the form of (8):

14 $$\begin{array}{l}{(W+{W}_{a}\nabla {\mathfrak{J}}{\bf{E}})}^{T}{\bf{v}}=\frac{\partial Q}{\partial {\bf{s}}}.\end{array}$$

Because of the non-linearity of the method, $\nabla {\mathfrak{J}}$ is not the direct differentiation of $\Im$. It results from the differentiation and reordering of the non-linear equations of the iterative method (equation (13)).

The inhomogeneous term in (14) can be obtained by differentiating equation (4). The focal plane is reached at a time *t* = *t*_*n*_ + *α* ⋅ *dt* between *t*_*n*_ and *t*_*n*+1_, with position and velocity *s* = *s*_*n*_ + *α* ⋅ (*s*_*n*+1_ − *s*_*n*_). Differentiating *Q* for a single particle results in

15 $$\begin{array}{ccc}\frac{{\rm{\partial }}Q}{{\rm{\partial }}{{\bf{s}}}_{{n}_{{\rm{f}}}}} & = & 2{[\begin{array}{c}\hat{{\bf{s}}}-{{\bf{s}}}^{\ast }\end{array}]}^{{\rm{T}}}[(1-\alpha )+\frac{{\rm{\partial }}\alpha }{{\rm{\partial }}{{\bf{s}}}_{{n}_{{\rm{f}}}}}{({{\bf{s}}}_{{n}_{{\rm{f}}}+1}-{\bf{s}}}_{{n}_{{\rm{f}}}})]\\ \frac{{\rm{\partial }}Q}{{\rm{\partial }}{{\bf{s}}}_{{n}_{{\rm{f}}}+1}} & = & 2{[\begin{array}{c}\hat{{\bf{s}}}-{{\bf{s}}}^{\ast }\end{array}]}^{{\rm{T}}}[\alpha +\frac{{\rm{\partial }}\alpha }{{\rm{\partial }}{{\bf{s}}}_{{n}_{{\rm{f}}}+1}}{{\bf{s}}}_{{n}_{{\rm{f}}}+1}]\end{array}$$

with the remaining values of $\frac{\partial Q}{\partial {\bf{s}}}$ being zero. For a beam of several particles, we apply the chain rule.

We can then calculate the adjoint system and reach the design sensitivities with

16 $$\begin{array}{c}\frac{{\rm{d}}Q}{{\rm{d}}{E}_{i}}={{\bf{v}}}^{T}[\,-\,{W}_{a}{\rm{\Im }}{\bf{1}}]\end{array}$$

where **1** is a vector of all ones and length of **E**.
